# Stratified Bacterial Diversity along Physico-chemical Gradients in High-Altitude Modern Stromatolites

**DOI:** 10.3389/fmicb.2017.00646

**Published:** 2017-04-12

**Authors:** Diego M. Toneatti, Virginia H. Albarracín, Maria R. Flores, Lubos Polerecky, María E. Farías

**Affiliations:** ^1^Planta Piloto de Procesos Industriales y Microbiológicos, Centro Científico Tecnológico – Consejo Nacional de Investigaciones Científicas y TécnicasSan Miguel de Tucumán, Argentina; ^2^Facultad de Ciencias Naturales e Instituto Miguel Lillo, Universidad Nacional de TucumánSan Miguel de Tucumán, Argentina; ^3^Centro Integral de Microscopía Electrónica, Centro Científico Tecnológico – Consejo Nacional de Investigaciones Científicas y Técnicas, Universidad Nacional de TucumánSan Miguel de Tucumán, Argentina; ^4^Department of Earth Sciences – Geochemistry, Utrecht UniversityUtrecht, Netherlands

**Keywords:** extremophiles, high-altitude lakes, stromatolites, 16S rRNA amplicon sequencing, UV radiation

## Abstract

At an altitude of 3,570 m, the volcanic lake Socompa in the Argentinean Andes is presently the highest site where actively forming stromatolite-like structures have been reported. Interestingly, pigment and microsensor analyses performed through the different layers of the stromatolites (50 mm-deep) showed steep vertical gradients of light and oxygen, hydrogen sulfide and pH in the porewater. Given the relatively good characterization of these physico-chemical gradients, the aim of this follow-up work was to specifically address how the bacterial diversity stratified along the top six layers of the stromatolites which seems the most metabolically important and diversified zone of the whole microbial community. We herein discussed how, in only 7 mm, a drastic succession of metabolic adaptations occurred: i.e., microbial communities shift from a UV-high/oxic world to an IR-low/anoxic/high H_2_S environment which force stratification and metabolic specialization of the bacterial community, thus, modulating the chemical faces of the Socompa stromatolites. The oxic zone was dominated by Deinococcus sp. at top surface (0.3 mm), followed by a second layer of Coleofasciculus sp. (0.3 to ∼2 mm). Sequences from anoxygenic phototrophic Alphaproteobacteria, along with an increasing diversity of phyla including Bacteroidetes, Spirochaetes were found at middle layers 3 and 4. Deeper layers (5–7 mm) were mostly occupied by sulfate reducers of Deltaproteobacteria, Bacteroidetes and Firmicutes, next to a high diversity and equitable community of rare, unclassified and candidate phyla. This analysis showed how microbial communities stratified in a physicochemical vertical profile and according to the light source. It also gives an insight of which bacterial metabolic capabilities might operate and produce a microbial cooperative strategy to thrive in one of the most extreme environments on Earth.

## Introduction

Microbial stromatolites are laminated organo-sedimentary structures accreted as a result of trapping, binding and/or *in situ* precipitation of minerals linked to the metabolic activities of microorganisms (Walter, 1976; Burne and Moore, 1987). They are regarded as the earliest complex ecosystems on Earth and although their emergence is still a matter of debate, geological records suggest biogenic signatures from about 3.5 billion years ago (Schopf and Packer, 1987; Schopf, 2006). Stromatolites were ubiquitous until the earliest Phanerozoic when their abundance rapidly declined due to the incipient grazing and borrowing activities of metazoans and protistans (Walter and Heys, 1985; Awramik and Sprinkle, 1999). Modern analogs of stromatolites are scarce, with very well-studied cases in the marine settings of Bahamas (Reid et al., 1995), in Shark Bay, Australia (Playford and Cockbain, 1976) and in Yellowstone Park, USA (Walter et al., 1972; Berelson et al., 2011).

Recently, we reported non-lithified modern stromatolites growing at the shore of the remote volcanic lake Socompa at 3570 m *a.s.l*. in the Puna (Argentinean Andes) (Farías et al., 2011a,b, 2013). This region is a desert area that withstand the most elevated doses of global solar radiation on Earth (Piacentini et al., 2003; NASA SSE Release, 2014). Monthly average of daily insolation reaches 6.6 KWhm^-2^ per day, a value that is within the largest in the world (Duffie and Beckman, 2013). UV Index (UVI) is likewise high at the Puna; Piacentini et al. (2004) calculated the average UVI for Argentina regions on December, 2011 and determined a UVI of 20 for La Quiaca (22.11° S, 65.57° W, 3,459 m) and only eight for the southern Argentinean city of Ushuaia (54.9° S, 68.3° W, 14 m). The Puna values are clearly above the ones detected at the Tibetan plateau (29.7° N, 91.1° E, 3,648 m), with monthly mean UVI over 16 in July (Ren et al., 1999). UV-light is not the sole source of stress for Socompa’s stromatolites; chemical stress is also present (Ruggieri et al., 2010); the lake water is alkaline (pH 9), with salt concentrations of about 10% (w/v) and arsenic content of up to 18.5 mg L^-1^ (Farías et al., 2013). However, favorable conditions are provided by a nearby hydrothermal stream that supplies nutrients and maintains the water column temperature at a relatively stable level (around 26°C).

Socompa’s stromatolites were characterized in terms of their mineral fraction and their bacterial bulk diversity. The mineral fraction was composed primarily of silicate due to high abundance of diatom frustules, and aragonite, most likely of biogenic origin and precipitated *in situ*. The microbial community was dominated by Proteobacteria (34%) Spirochaetes (8%), *Deinococcus*-*Thermus* (7%), Bacteroidetes (6%), Firmicutes (5%), Cyanobacteria (3%), Chloroflexi (1%), and 33% of unclassified sequences. Interestingly, pigment and microsensor analyses performed through the different layers of the stromatolites (50 mm-deep) showed steep vertical gradients of light oxygen, hydrogen sulfide and pH in the porewater. Given the relatively good characterization of these physico-chemical gradients (Farías et al., 2013), the aim of this follow-up work was to specifically address how the bacterial diversity stratified along the top six layers of the stromatolites which seems the most metabolically important and diversified zone of the whole microbial community. In only 7 mm, microbial communities shifted drastically from a UV-high/oxic to IR-low/anoxic/high H_2_S conditions forcing the spatial compartmentalization of diverse taxonomic groups, and the concomitant functional specialization of each layer. The putative metabolic functions of these stratified bacterial community and their influence on the chemical faces of the Socompa stromatolites is likewise discussed.

## Materials and Methods

### Sample Collection

Samples analyzed in this study were collected at noon in February of 2011 (austral summer) in parallel to the samples analyzed by Farías et al. (2013). Permission for sample collection was granted by the Ministerio de Ambiente y Desarrollo Sustentable, Salta, Argentina (number 000388; 17–09–2010).

**FIGURE 1:**
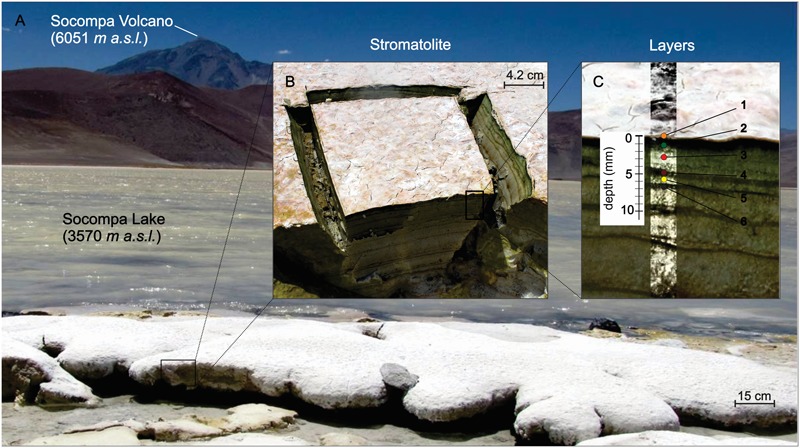
**Environmental setting of Socompa’s Stromatolites. (A)** Sampling site at the shore of the alkaline Socompa Lake showing domical and columnar stromatolite morphologies. **(B)** Transversal cut of the sample with clear visible laminations. **(C)** Magnification on stratification showing a pinkish-white layer in the surface (ly1, orange dot), a dark green layer (ly2, green dot), a light brownish (ly3, red dot), a dark brownish layer (ly4, brown dot), a light brownish (ly5, yellow dot), and a dark brown layer (ly6, dark brown dot).

Columnar round-dome shaped stromatolites are found at the southern shore of the Socompa Lake, where a stream of hydrothermal water enters the lake. Unlike in the winter, when the stromatolites are covered due to high snowfalls, during summer, higher evaporation rates exposes the top 10–30 cm to dry air and direct sunlight (**Figure 1A**). Top six layers of the sample, reaching from the surface down to a depth of 7 mm, were dissected by a sterile scalpel based on their distinct coloration (**Figures 1B,C**): the top layer (0–0.3 mm depth) was white with little cracks and pinkish patches; the second layer (0.3–1.5 mm) was dark-green; the color of the subsequent layers, whose thickness varied with depth, changed between light and dark brownish.

Samples for DNA and pigment analyses were frozen in liquid nitrogen, stored in the dark, and processed within a week. Samples for SEM-EDS were stored in the dark at 4°C, fixed at the lab and processed within 1–2 weeks.

### Scanning Electron Microscopy

Samples for SEM-EDS were fixed over night at 4°C in a Karnovsky fixative comprising formaldehyde (8% v/v), glutaraldehyde (16% v/v), and phosphate buffer (pH 7). The fixed samples were washed three times with phosphate buffer and CaCl_2_ for 10 min, and fixed with osmium tetroxide (2% v/v) over night. Afterward, the samples were washed twice with ethanol (30% v/v) for 10 min, placed in stabs, dried at a critical point, and sputtered with gold. Specimens were observed under vacuum using a Zeiss Supra 55VP (Carl Zeiss NTS GmbH, Germany) scanning electron microscope coupled with an energy dispersive X-ray detector (INCA, Oxford Instruments).

### DNA Extraction, PCR Amplification, and 454 Pyrosequencing

Three replicates of each subsampled layer were collected. DNA of each replicate (wet weight of ∼0.2 g) was extracted using the Power Biofilm^TM^ DNA Isolation Kit (MO BIO Laboratories, Inc.) along with a bead tube method and the inhibitor removal technology^®^(IRT) according to the manufacturer’s instructions. Quality of each DNA was tested by PCR amplification of the 16S rDNA.

Bacterial 16S rRNA gene sequencing was done at the INDEAR genome sequencing facility, briefly: the V4 hyper-variable region of the Bacterial 16S rRNA gene was amplified using the universal primers suggested by the Ribosomal Database Project (RDP^1^). The primers contained the Roche 454 sequencing A and B adaptors and a 10 nucleotide “multiple identifier” (MID). PCR amplification was done on a FastStart High Fidelity PCR system (Roche Applied Science, Mannheim, Germany) following the manufacturer’s instructions. Five independent PCRs were performed to reduce bias. Two negative controls with no template were also performed. The PCR conditions were 95°C for 5 min, followed by 30 cycles of 95°C for 45 s, 57°C for 45 s and 72°C for 60 s, and a final elongation step at 72°C for 4 min. The five reactions were pooled, purified and sequenced on a Genome Sequencer FLX (Roche Applied Science) following the amplicon sequencing protocol provided by the manufacturer. A total of 116227 filtered sequences with an average length of 250 bp were obtained for the six layers analyzed. Filter parameters were set to reject reads that had mean quality score < 25, maximum homopolymer run > 6, number of primer mismatches > 0, and read length < 200 bp. The sequences were deposited as FASTAQ in the NCBI Sequence Read Archive (SRA) under the accession number of the bioproject ID PRJNA281688^2^.

### Taxonomy and Diversity Analysis along Depth Profile

Taxonomic assignation of the 16S pyrotags was performed using the QIIME software v1.7.0 (Caporaso et al., 2010). Sequences were aligned with the Pynast module included in QIIME using Silva database release 108 non-redundant template for QIIME^3^. Gap-only sites of the resulting alignment were eliminated. The 116227 16S rRNA (HV4) pyrosequences obtained from all layers were clustered into operational taxonomic units (OTUs) with UCLUST at 0.97 and 0.80 identities, and their abundances were normalized at the lowest number of sequences in the compared datasets (17653). 230 clusters were obtained. *Mitochondria* and *Chloroplast* classified clusters were eliminated. Classification of the 0.97 identity OTUs was carried out against the Greengenes database using the RDP classifier included in QIIME (bootstrap confidence of 50%). Alpha diversity metrics were calculated using QIIME. Since most of the clusters occur at very low relative abundances, the representation was simplified, as only 29 contained almost ∼99% of all sequences, thus, allowing us to include most of the dataset with only 29 clusters. In order to assign a taxonomic representative to each cluster, every sequence within a cluster was classified against the SSU Ref_99_NR database. The best hit (>97% identity) for each sequence was selected of the megablast alignments (NCBI-blast+), and the number of identical hits were counted and listed. Alignments of the OTUs sequences were visually inspected in MEGAN 5 software (Huson et al., 2011). The most abundant representatives were chosen to assign taxonomy to each cluster. **Supplementary Figure S2** lists the relative amount of the most abundant sequences in each cluster together with taxonomy.

The relatives abundances of OTUs and distribution on a heat-map were designed from the OTUs table obtained in QIIME and using R statistical software (R Core Team, 2014). The clusters and layers dendrograms were created with the Bray–Curtis method using “heatmap.2” (Warnes et al., 2015) in R statistical software.

## Results

### Bacterial Richness and Coverage

**FIGURE 2:**
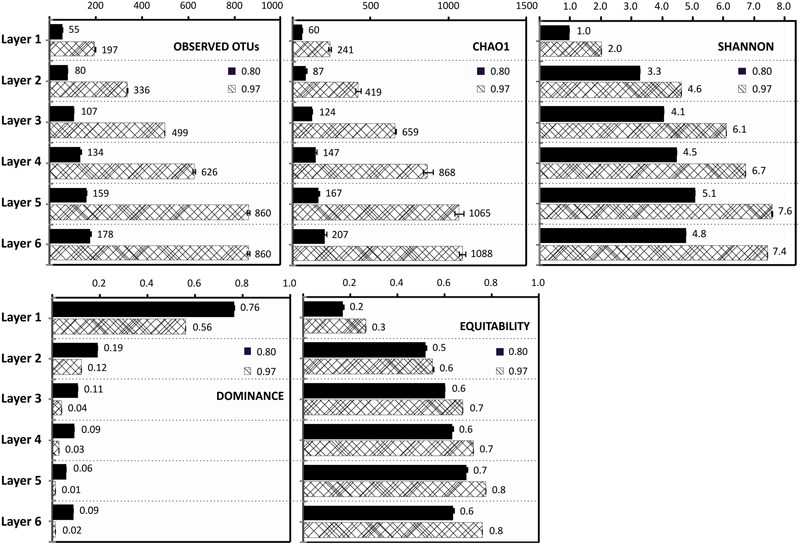
**Diversity and Dominance Indexes.** Observed OTUs, Chao 1 estimator, evenness, dominance, and Shannon indexes for the six layers, at 97 and 80% OTU identity, normalized with the number of sequences of the smaller dataset (17653).

Rarefaction analysis performed with sequences at 97% identity and at 80% gave 1,670 OTUs and 231 OTUs, respectively. None of the rarefaction curves at 97% reached an asymptote, indicating the numbers of OTUs (97%) were underestimated (**Supplementary Figure S1**). The observed OTUs, clustered at 80% identity, were almost the same as predicted by the Chao1 estimator, indicating that the dataset virtually covers the abundance of phyla in the samples (**Figure 2**). Rarefaction analysis also showed how the number of OTUs increased with sampled layer depth, in fact the dominance and evenness indicators reveal a simple community with dominant OTUs at the top layer, and a complex community with more evenness increasing with depth (**Figure 2**). Shannon index ranged from 0.8 at surface to almost 8 at 7 mm depth, indicating an abundant diversity of species in deeper layers (**Figure 2**).

### Layer-by-layer Bacterial Diversity

**FIGURE 3:**
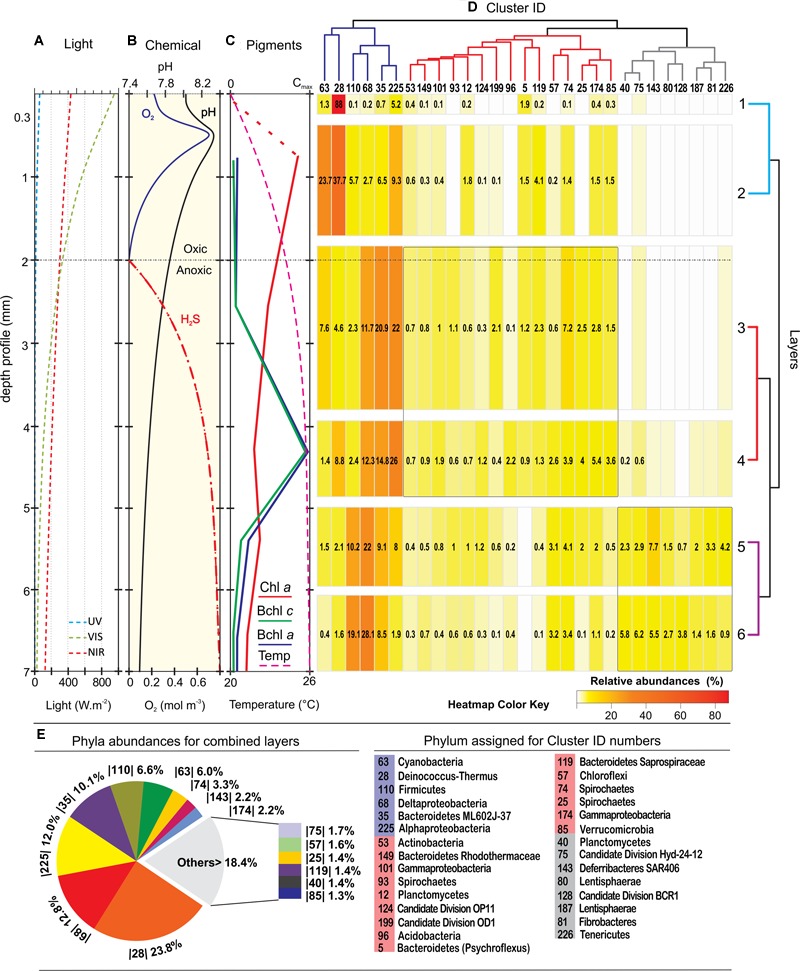
**Depth profiles of physico-chemical parameters and bacterial diversity. (A)** Light profile; **(B)** oxygen, pH, and H_2_S profile; **(C)** pigment profile adapted from Farías et al., 2013). **(D)** Heatmap of the 29 most abundant clusters of sequences; **(E)** phyla abundances for the combined layers. The clusters, represented in a dendrogram according to their pattern of distribution, displays three main branches; the dark-blue branch with key functional groups that were present virtually in all layers, although they clearly concentrate in great abundance at three different levels; the red branch showing clusters that were mostly widespread in the middle; and the gray branch with clusters located at dark anoxic layers. The phylum assignation of the clusters ID are listed down, and the ‘best hit’ taxonomic assignations are listed in the **Supplementary Figure S2**. Relative abundances (>0.1%) in the heatmap are indicated within the boxes in numbers and with a color key. A dendrogram of the layers shows pairs that match according to their different biological and physicochemical microenvironment.

Vertical physico-chemical profile detected through microsensor measurements and pigment analyses (Farías et al., 2013) defined three differential zones in the stromatolite; an oxygen rich zone (0–2 mm) where UV light is intense at the first 0.3 mm and Chl a reaches its maximum; a transitional zone from micro-oxic to anoxic with low-H_2_S (2–5 mm) dominated by IR-light and Bchl a/c pigments; and finally an IR-low, anoxic and high-H_2_S zone between 5 and 7 mm. The bacterial sequences from each of the seven layers obtained in this work were correlated, plotted against these vertical physico-chemical gradients and clustered in three major branches according to their distribution and abundance (**Figures 3A–D**). When sequences for all layers were analyzed together, dominant groups (**Figure 3E**) were Proteobacteria (27%) and *Deinococcus-Thermus* (23.8%). In less proportion we found Bacteroidetes (11.5%), Firmicutes (6.6%), Cyanobacteria (6%), Spirochaetes (4.7%), Deferribacteres (2.2%), Chloroflexi (1.6%), Planctomycetes (1.4%), Verrucomicrobia (1.3%) and Candidate Divisions.

Although the results from the analyses of the partial 16S rDNA sequences were correlated by data from microsensors and pigments (Farías et al., 2013), assignations must have been taken carefully since only nearly complete 16S rRNA sequences give accurate measures of taxonomic diversity (Yarza et al., 2014). Rare phyla, candidate division and sequences derived from uncultured organisms imply that other methods of sequencing, with at least full length 16S rDNA analysis, had to be made to accomplish a better characterization. Considering the profiles and our previous results (Farías et al., 2013), it is expected that more unclassified sequences come into sight with depth.

### Oxic, Photic, and UV-stressed Zone

Two very well-defined microbial communities inhabited layer 1 (top 0.3 mm) and layer 2 (up to 2 mm; **Figure 5**). The distribution of taxonomical groups was similar for both layers although the relative abundance of each taxa varied substantially between them (**Figure 3D**). The most abundant groups fell within the blue branch of the heat-map while the rest stayed within the red branch.

**FIGURE 4:**
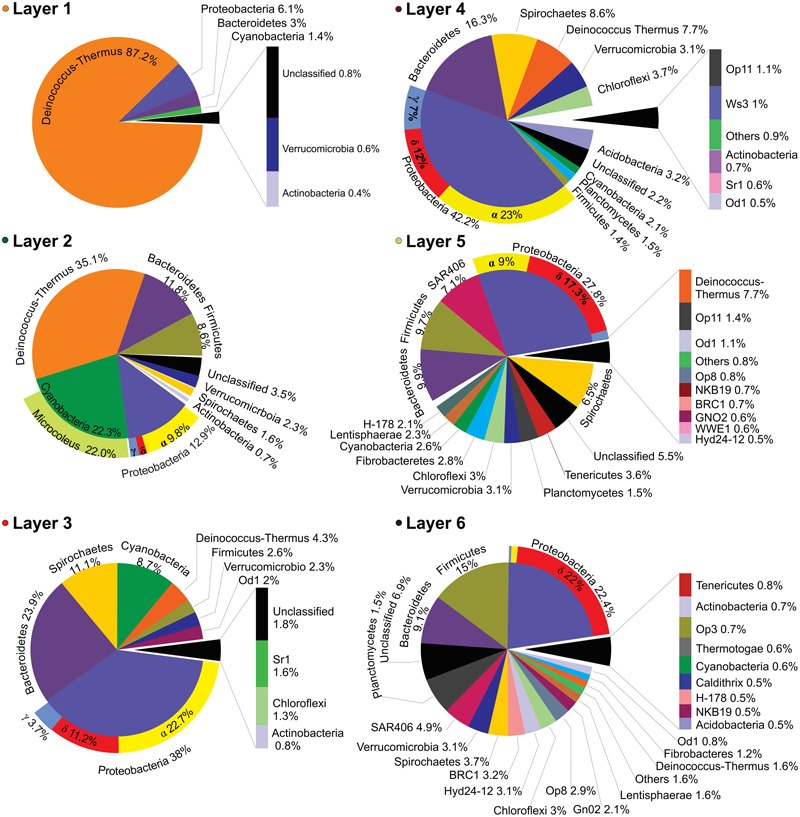
**Layer-by-layer taxonomical profile.** Bacterial taxonomic assignations of all six layers expressed in percentage of the relative abundances obtained from the 16S (HV4) pyrotags clustered at 97% OTU identity level and classified against the green-genes database. Only OTUs with values ≥ 0.4% were plotted.

Layer 1 (**Figure 4**) was dominated by *Deinococcus-Thermus* (87.2%). Other sequences from the top layer came from likely UV-sensitive microbes and micro-aerophiles that have temporally migrated to the surface during night (Albuquerque et al., 2005; Villanueva et al., 2007). The rest of OTUs were assigned to Proteobacteria (6.1%), Bacteroidetes (3%), and Cyanobacteria (1.4%) with little contribution of groups like Verrucomicrobia and Actinobacteria (0.6 and 0.8%, respectively). It was not possible to classify ca. 1% of the sequences. Layer 2 presented a more even representation of same phyla found in layer 1 (**Figure 4**), with the major groups being *Deinococcus-Thermus* (35.1%), Cyanobacteria (22.3%), Proteobacteria (12.9%), Bacteroidetes (11.8%), and Firmicutes (8.6%). Other OTUs were assigned to Verrucomicrobia, Spirochaetes, and Actinobacteria (2.3, 1.6, and 0.7%, respectively). A significant proportion of the sequences (3.5%) remained unclassified.

**FIGURE 5:**
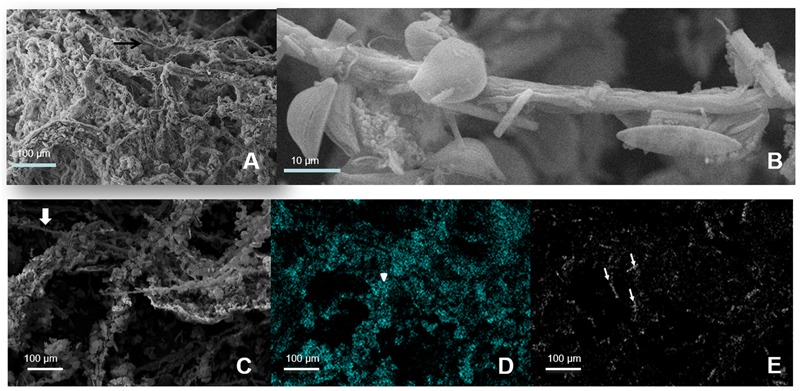
**Scanning electron microphotography of layer 2. (A)** Filamentous Cyanobacteria. **(B)** Magnified view of one filamentous with attached carbonate crystals and diatoms. **(C–E)** EDS images from the filamentous Cyanobacteria green layer with diatoms and crystals attached. Gray: Original Image 600X; Cyan: Silicium, representing diatoms; White: Calcium, representing aragonite crystals. Thick arrows: Cyanobacteria filaments; Point arrows: diatoms; Thin arrows: aragonite crystals.

*Cyanobacteria* and *Deinococcus*-*Thermus* were therefore the dominant groups at the oxic zone (blue branch of the heatmap, **Figure 3D**). Nevertheless, they appeared in all analyzed layers spreading out to even anoxic and IR-low layers 6–7 (**Figure 4** and **Supplementary Figure S2**). SEM/EDS (**Figure 5**) confirmed the presence of filamentous cyanobacterial sheaths tightly bound to diatoms and mineral micro-crystals immediately beneath the layer of *Deinococcus*, ranging from 0.3 to ∼2 mm depth. In accordance, pyro-sequencing indicated that the layer 2 had almost ∼25% of the sequences of the mat builder *Coleofasciculus* sp.

*Cyanobacteria* were mainly represented by sequences related to the cosmopolitan mat-builder *Coleofasciculus chtonoplastes*, specifically to strain PCC 7420 first described in the stromatolites of Shark Bay, Hamelin Pool Domal samples (Papineau et al., 2005). In turn, the sequences assigned to the *Deinococcus-Thermus* fit almost exclusively within the genus *Truepera* (**Supplementary Figure S2**). Nearly all sequences of the cluster 28 were 100% identical to an uncultured *Deinococcus* also found in environmental samples from Guerrero Negro hypersaline mat-10, from an altitude of 310 m *a.s.l*. (sample depth 1 m below water level, 34–49 mm depth into the mat) (Harris et al., 2012). Bacteroidetes, Firmicutes, Spirochaetes, and Actinobacteria sequences gave higher identity with uncultured microorganisms. Within Proteobacteria, sequences were assigned to Deltaproteobacteria, Alphaproteobacteria, and Gammaproteobacteria.

### Transitional Zone: IR-dominated with Micro-oxic to Oxic Conditions and Low H_2_S

Layers 3 and 4 were distinguished by an increasing number of phyla, and intra-phylum diversity with a growing number of OTUs reaching 97% compared to 80% in layers 1 and 2 (**Figure 2**). The clusters of these central layers fall also in the blue and red branch of the heatmap (**Figure 3D**).

Central layers presented a more even representation of diverse phyla (**Figure 4**) i.e., Proteobacteria (38 and 42.2%), Bacteroidetes (23.9 and 16.3%), and Spirochaetes (11.1 and 8.6%). Within Proteobacteria, anoxygenic phototrophs from alphaproteobacteria were prevalent while the more abundant *Bacteroidetes* were closely related to uncultured environmental sequences of *Cytophagia* Class (**Figure 3D**) and to clone ML602J-37 from environmental samples of the anoxic monimolimnion (35 m) of the arsenic rich soda lake, Mono Lake (Humayoun et al., 2003). Unlike in the oxic zone, Cyanobacteria (8.7 and 2.1%) and *Deinococcus-Thermus* (4.3 and 7.7%) were minor groups. The rest of OTUs in both layers -accounting for less than the 5% each- corresponded to Firmicutes, Verrucomicrobia, Chloroflexi, Acidobacteria, Planctomycetes, and Actinobacteria. Many sequences were assigned to candidate divisions such as OD1 and OP11. Less proportion of the sequences (1.8%), with respect to the oxic zone, remained unclassified.

Interesting to note is that from layer 3 and on, a shift in the proportion of Deltaproteobacteria (DP) is observed (**Figure 4**). For instance, in layer 2, DP represented around 1% of the total Proteobacteria while in layers 3 and 4 it reached ca. 12%, reaching a final proportion of 17 and 22% in layers 5 and 6, respectively. The main group representing DP in the stromatolites are the sulfate reducing bacteria (SRB) Desulfobacterales from the Desulfobactereaceae family, with most of the hits lying within the genus *Desulfotignum*. The DP increase along depth is coincident with the rise in hydrogen sulfide production in the stromatolite profile (**Figure 3B**).

### Anoxic, IR-Low, High-H_2_S Zone

Apart from some groups clustered in the blue and red branch of the heat-map, deeper layers 5 and 6 were occupied by clusters contained in the gray branch (**Figure 3D**). Proteobacteria (27.8 and 22.4%), Firmicutes (15 and 9.7%) and Bacteroidetes (9.9 and 9.1%), were the dominant group with the rest of the phyla distributed in less proportions: SAR406 (7.1 and 4.9%), *Deinococcus-Thermus* (7.7 and 1.6%), Spirochaetes (6.5 and 3.7%), Tenericutes (3.6 and 0.6%), Verrucomicrobia (3.1%), Chloroflexi (3%), Fibrobacteres (2.8 and 1.2%), Cyanobacteria (2.6 and 0.6%), Lentisphaerae (2.3 and 1.6%), and Planctomycetes (1.5%). The rest of sequences lied in groups that accounted for less than 1% each: i.e., Actinobacteria, Thermotogae, Acidobacteria, and several candidate divisions. In addition, it was observed a high diversity of uncultured bacteria belonging to the candidate divisions BRC1, Hyd-24 and OP8, and sequences from Firmicutes, Tenericutes, Fibrobacteres, SAR406 and the PVC superfamily, an amalgamation of species from the phyla Planctomycetes, Verrucomicrobia, and Chlamydiae, along with the Lentisphaerae and other candidate divisions (**Figure 4**). Firmicutes found in these deep-layers included mainly strictly anaerobes of Clostridiales and Lactobacillales (**Supplementary Figure S2**). A large proportion of sequences remained unclassified (5.5 and 6.9%).

## Discussion

Dominant groups found in the stromatolites were Proteobacteria and *Deinococcus-Thermus*. In accordance, preliminary work indicated that Proteobacteria was predominant in a 50 mm-deep sample of same stromatolite (Farías et al., 2013). Based on the 16S rRNA gene sequences, Socompa stromatolites bulk diversity can be compared to other microbial mats from the Puna-High Andes region such as La Brava (Farías et al., 2014) and Llamará (Rasuk et al., 2014) in which, Proteobacteria and Firmicutes are the predominant taxa. On the other hand, Socompa’s diversity seems quite different from low-altitude marine stromatolites with less-stressing environmental conditions (Papineau et al., 2005; Baumgartner et al., 2009); the Shark Bay stromatolites are dominated by Alphaproteobacteria, Actinobacteria, and Cyanobacteria while the Highborne Cay stromatolites’s main taxa are the Alphaproteobacteria and Cyanobacteria. Yellowstone hot-spring’s siliciclastic stromatolites (Berelson et al., 2011; Pepe-Ranney et al., 2012) displayed Cyanobacteria and *Deinococcus-Thermus* as predominant groups. Modern freshwater microbialite ecosystems located at Ruidera Pools National Park in Central Spain and at the Cuatros Ciénegas Basin in Mexico have as dominant taxa Cyanobacteria and Firmicutes (Breitbart et al., 2008; Santos et al., 2010).

Layer-by-layer 16S rRNA amplicon sequencing compared with physico-chemical data clearly showed how microbial communities shifted drastically from a UV-high/oxic to IR-low/anoxic/high H_2_S conditions forcing compartmentalization and functional specialization in only 7-mm. The top layers are oxic and are the most affected by the highest UV-stress incident on the stromatolites surface, thus selecting UV-resistant microbes. In accordance, the top 0.3-mm layer contained a low microbial diversity dominated by *Truepera* (*Deinococcus-Thermus)*. *Deinococcus* and *Truepera* strains are known as highly radio-resistant microbes (Makarova et al., 2001; Ivanova et al., 2011) and as such in the stromatolites they may act as a UV-shield, preventing direct sunlight to affect lower photosynthetic layers. *Deinococcus* are able to screen UV light due to the presence of a special pigment called as deinoxanthin, which may explain the pinkish color on the surface of the stromatolite (Farías et al., 2013). Likewise, in the Shark Bay stromatolites, the uppermost layer had a distinctive black coloration due to the presence of a UV-quenching pigment scytonemin (Goh et al., 2009), likely produced by *Gloeocapsa* sp., a unicellular cyanobacterium (Dupraz et al., 2004). In the top layers of microbialites and microbial mats of La Brava (2300 m *a.s.l*.) *Deinococcus* is the predominant microbe reaching up to 4 and 18%, respectively (Farías et al., 2014; Rasuk et al., 2014). Not only the high UV radiation at the HAAL can explain such abundance of *Deinococcus-Thermus* phylum, but also they might be playing an important role in the stromatolite/mat arsenic metabolism (Gihring and Banfield, 2001; Rhine et al., 2007). Alternatively, their abundant occurrence in the first layer may be due to photoreceptors able to sense and respond to different qualities of light. In *Deinococcus radiodurans*, the bacteriophytochrome photoreceptor (BphP) which absorbs red and far-red light was already studied (Davis et al., 1999). It acts as a protein kinase that begins light sensing by phosphorylating other proteins which then stimulates the bacterium to produce carotenoids, pigments typically used to protect from high light conditions.

Beneath the first layer, an intricate net of cyanobacterial *Coleofasciculus* sp. sheaths coupled to diatoms and some Bacteroidetes were observed. A large amount of *Truepera* sequences were also registered at the second layer which can correspond to indigenous microbes or due to random contamination from the first one considering the thinness of this layer. Alternatively, a high amount of *Truepera* extracellular DNA from the first layer may be present in the underneath cyanobacterial extracellular polysaccharides (EPSs) matrix, due to UV-induced sheering caused by high solar irradiation on the top (Vlassov et al., 2007; Pietramellara et al., 2009; Wang et al., 2015). In any case, this second layer is most likely responsible for the oxygenic photosynthesis, EPS matrix production and probably for the main carbon and nitrogen fixation process as reported for other stromatolites (Dupraz et al., 2004; Breitbart et al., 2008; Goh et al., 2009; Santos et al., 2010). As the rule, Cyanobacteria were described as the predominant organisms present in all modern stromatolites driving the metabolic force behind stromatolitic mat metabolism and early lithification (Foster and Green, 2011). Nevertheless, in this work we found that Cyanobacteria account for only the 6% of taxa. This low cyanobacterial diversity which is also the case for previous reports on HAAL’s microbial mats (Farías et al., 2013, 2014; Rasuk et al., 2014) may be explained by the low occurrence of these microbes at the HAAL. Most probably due to their high sensitivity to environmental stresses such as UV, high salt, and arsenic (Cabrol et al., 2009; Albarracín et al., 2015). The few OTUs for cyanobacteria in the stromatolite -mainly assigned to *Coleofasciculus* and uncultured bacteria- are much more abundant in the photic, upper zone but also present in deeper parts of the ST. This dual allocation might be explained by negative phototaxis, as migration of cyanobacteria just beneath the *Deinococcus-Thermus* photoprotective layer upon changes of light conditions was demonstrated before (Farías et al., 2013). On the other hand, *Coleofasciculus* is equipped with two photosystems that can use H_2_O to carry out a plant-like oxygenic photosynthesis even under anoxic conditions and also are able to use H_2_S in anoxygenic photosynthesis producing thiosulfate as the end product of sulfide oxidation. More important, they are adapted to perform anoxygenic photosynthesis simultaneously with oxygenic photosynthesis in the presence of sulfide (de Wit and Van Gemerden, 1987), a property that is an advantage in this particularly sulfide-rich environment and might be an explanation of the abundances of sequences at deeper layers.

Central layers may be specialized in organic matter and EPS degradation by fermenting and producing low-molecular organic acids and alcohols as observed for other similar microbialites (Dupraz et al., 2004). The finding of about 15–20% of the sequences corresponding to *Bacteroidetes*, which has a documented ability to convert complex polysaccharides into useable low molecular weight compounds (LMWC), support this hypothesis. *Bacteroidetes* can be found involved in many important metabolic activities in the nature and human gut including fermentation of carbohydrates, utilization of nitrogenous substances, and biotransformation of bile acids and other steroids (Thomas et al., 2011). Most of these bacteria are saccharolytic, which means that they obtain carbon and energy by hydrolysis of carbohydrate molecules. The main by-products of their anaerobic respiration are acetic acid, iso-valeric acid, and succinic acid. Acids by-products and LMWC causes a decrease in pH and redox potential, allowing sulfate to be reduced and stimulating the SRB (Dupraz et al., 2004).

Away from UV exposure, IR is the main source of photons for layers 3–7 (**Figure 3A**), indicating that these layers are also photosynthetically active. Because of the strong competition for infrared light, an effective absorption in this wavelength range would constitute a selective advantage for survival and proliferation in the central layers. *Alphaproteobacteria* -11% of all dataset- from the *Rhodobacteraceae* family found beneath cyanobacterial bundles and able to carry out anoxygenic photosynthesis may act as the main bacterial group using near-IR light at layers 3 and 4. The most prominent genera detected within were *Roseovarius, Rhodovulum*, and *Roseibaca*. These genera were reported in many different marine habitats or high-altitude alkaline lakes, e.g., 35% of the sequences of the ID 225 gave a hit with environmental sequences of *Roseovarius* from the Tibetean Xiaochaidan Lake (Zhang et al., 2013). It is likely that most of the peak of Bchl *a* at 4.5 mm depth (Farías et al., 2013) was derived from these purple photosynthetic bacteria, as also Bchl *c* peak from the green sulfur bacteria *Chloroflexi*. The photosynthetizers of central layers may also comprise cyanobacteria migrating from upper layers and diatoms, according to pigment analyses and electron microscopy (**Figure 3C**, Farías et al., 2013). This is not surprising as diatoms can survive in dark sulfidic marine sediments for many years (Ribeiro et al., 2011). Apart from being in charge of the primary production, the role of diatoms in these layers can be to accumulate nitrate (Kamp et al., 2011), thus cycling nitrogen, or to supply organic matter to SRB. In addition, central layers comprised a vast number of uncultured bacteria in a low frequency but it is uncertain which physiological roles they play in the stromatolites.

Interestingly, the most novel microbial community was found 6-mm beneath the surface within IR-low, H_2_S-rich, anoxic layers. *Deferribacteres* sequences in deeper layers might be an indication that higher temperatures and iron reduction prevails in deep anoxic layers. Another indication of the “hot” nature of these layers are the singular presence of members of the phylum *Caldithrix*; *Caldithrix abyssi* and *Caldithrix palaeochoryensis* are known as anaerobic, mixotrophic, thermophiles obtained from hydrothermal vent and sediment environments, respectively (Miroshnichenko et al., 2010). PVC members, abundant in these deeper layers, may have a likely role as quimiolithotrophs and oligotrophs. A group of *Planctomycetes* are able to perform anaerobic ammonium oxidation, an important function that might have implications in the sediment and atmosphere nitrogen cycle (van Niftrik and Jetten, 2012). Farías et al. (2013) indicated that two abundant distant phyla related to Verrucomicrobia and *Desulfotignum* bacteria were present in a sample of 50 mm-deep. In this work, *Desulfotignum*-related sequences were found from layer 3 and below, increasing their abundance with depth. These genus comprises to date only three species: *D. balticum, D. toluenicum*, and *D. phosphitoxidans*. The formeroxidizes phosphite to phosphate as its only source of electrons, with either sulfate or CO_2_ as electron acceptor to gain its metabolic energy, which is of exclusive interest (Poehlein et al., 2013). These mechanisms may be present also in the Socompa’s stromatolites taking in account the prevailing anoxic conditions in these layers. Nevertheless, Verrucomicrobia sequences were not much abundant, so it is assumed that they might inhabit deeper layers. A high dominance of the phyla Verrucomicrobia (44%) were found also in the near-by fumaroles (25°C) of the Socompa Volcano at 5,824 m *a.s.l*., which also resulted phylogenetically novel and affiliated with the subphylum Spartobacteria (Costello et al., 2009).

The Socompa stromatolite’s diversity thus, increased with depth not only in the number of phyla but also by broad intra-phylum diversity (**Figures 3, 4**). The stromatolite-water/air interface seems the most stressful zone of the stromatolites as in these layer the lowest diversity was observed. A likely explanation is that only a few taxa may cope with the prevailing steady-state conditions (high UV-irradiation, low availability of organic matter) together with the fluctuating settings more pronounced at the interface than at the inner stromatolite (chemical stress by salt and arsenic, seasonal submerged/exposed regime, mechanical disturbances such as winds, water movement, volcanic ashes and hydrothermal input, micro-invertebrates grazing). These plethora of “extremes” configure a unique ecological niche that may select metabolism types toward photo-resistance and probably phototrophy in the first millimeters. In fact, all three available genomes of strains isolated from the top layers of the stromatolites displayed a microbial rhodopsin-coding gene indicating the importance of this kind of strategy for survival (Farías et al., 2014; Gorriti et al., 2014; Albarracín et al., 2016). In contrast, the deeper zone although anoxic, with low pH and high-sulfur content, supported a higher diversity (**Figures 3, 4**). These microbial inner communities may endure more stable conditions while benefit from abundant and diversified carbon and nitrogen sources (e.g., alcohols, organic acids, carbonate, nitrates) and terminal electron acceptors for respiration (e.g., sulfate, nitrate, sulfur, CO_2_, ferric iron, arsenic). More work will be needed to elucidate bacterial specific metabolisms within each layer.

## Conclusion

High solar irradiation challenged microbial communities from High-Altitude Andean Lakes (HAAL) in many ways selecting UV resistance/adaptation phenotypes (Albarracín et al., 2012, 2014, 2016). Biofilms, microbial mats, and microbialites are widespread in most HAAL’s lakes (Albarracín et al., 2015), suggesting that this associative behavior pursues a common benefit to microbe survival. In this work, we presented Socompa stromatolites as test cases for understanding the spatial compartmentalization of diverse taxonomic groups in a “cooperative” microbial society, and the concomitant functional specialization of each layer. As microbialites thriving under a natural high-UV radiation regime, these stromatolites represent a modern analog of their counterparts to study process of microbialite formation in a close proxy environment to Early Earth. The elucidation of metabolic patterns associated with stromatolite formation and the study of specific metabolisms on As, UV-resistance and other extremes will then open a high window for better understanding the ecology, biogeochemistry and evolution of Precambrian analogs. A comprehensive metagenome study and the reconstruction and analysis of scaffold genomes within the stromatolites is underway to unravel this complex, yet underexplored microbial community.

Finally, we would like to state that given the uniqueness of the Socompa environment and the broad unknown microbial diversity they possess, protection policies and continuous monitoring should be undertaken to avoid the huge impact of nearby and current mining activities menacing the preservation of these microbial treasures.

## Author Contributions

DT, LP, and MEF designed and performed the research. MEF conceived the original HAAL project. DT, LP, and MRF performed HAAL sampling expeditions and provided the stromatolites. DT performed the experiments and bioinformatic data analysis. LP and MRF performed the microsensors and pigments analyses. VA and DT prepared, obtained and interpreted the SEM images. MEF, LP, and VA provided reagents/materials and analysis tools. VA, DT, and LP wrote the paper. MEF and VA revised the manuscript. MEF and VA obtained funding for the original project idea. All authors read and approved this manuscript.

## Conflict of Interest Statement

The authors declare that the research was conducted in the absence of any commercial or financial relationships that could be construed as a potential conflict of interest.

## Footnotes

1 http://pyro.cme.msu.edu/pyro/help.jsp

2 http://www.ncbi.nlm.nih.gov/bioproject/281688

3 http://www.arb-silva.de/no_cache/download/archive/qiime/

## Supplementary Material

FIGURE S1 **Alpha-diversity analysis.** Rarefaction plots of observed OTUs **(E)**, Chao 1 estimator **(A)**, evenness **(B)**, dominance **(C)**, and Shannon **(D)** indexes at 97 and 80% OTU identity, normalized with the number of sequences of the smaller dataset (17653).

FIGURE S2 **Taxonomic assignation of the clusters ID in the heatmap.** Clusters ID numbers along with the major taxonomic assignations (see Materials and Methods), the contribution rate for a given assignation in the cluster, and the total percentage of sequences in the cluster represented with those taxonomic assignations.
