# Supplementary figures and images for: Stratified Bacterial Diversity along Physico-chemical Gradients in High-Altitude Modern Stromatolites
Source: Front Microbiol. 2017 Apr 12;8:646. doi: 10.3389/fmicb.2017.00646 (PMC5388776; doi:10.3389/fmicb.2017.00646)

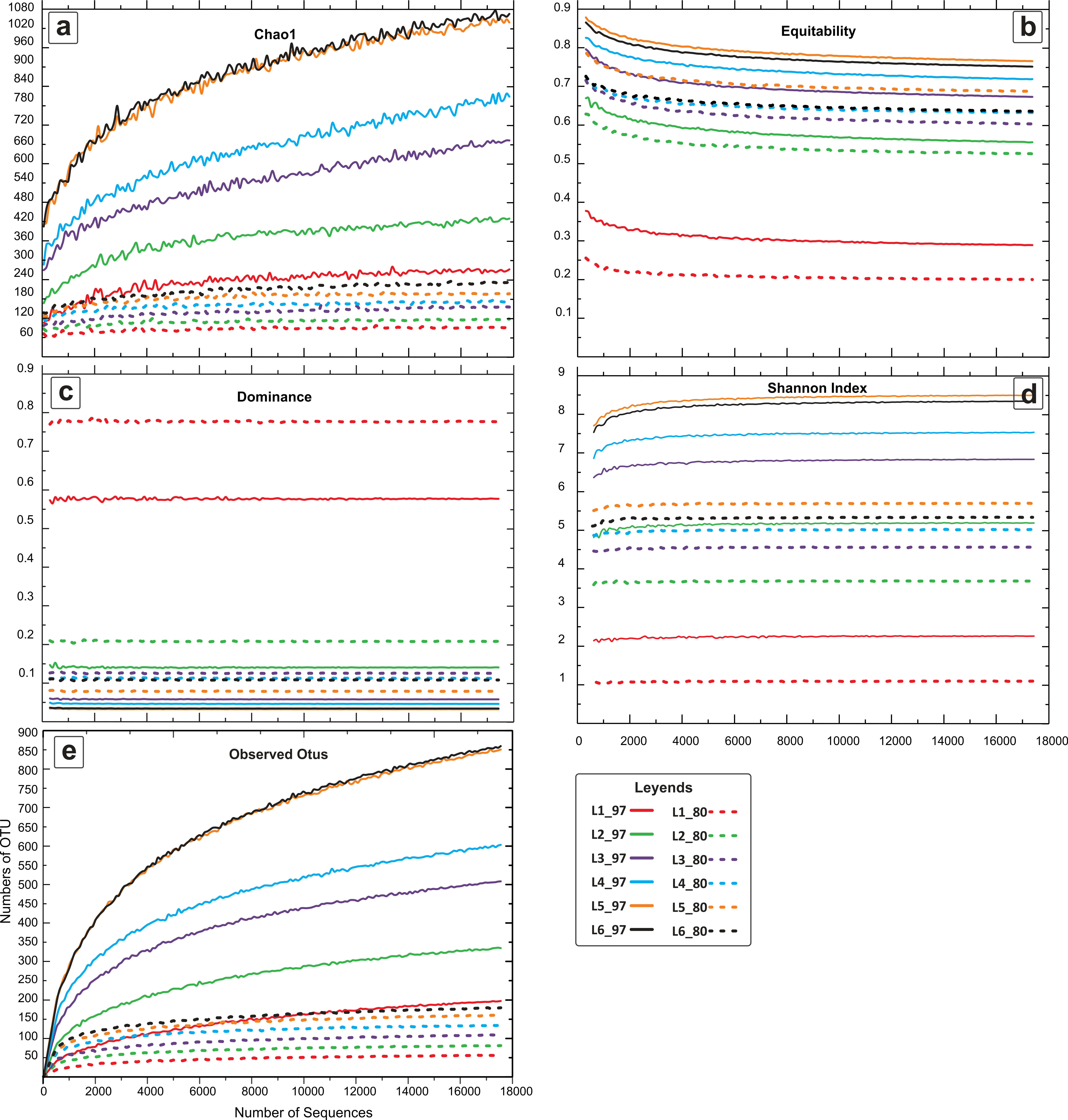

Supplement: FIGURE S1 — Alpha-diversity analysis. Rarefaction plots of observed OTUs (E), Chao 1 estimator (A), evenness (B), dominance (C), and Shannon (D) indexes at 97 and 80% OTU identity, normalized with the number of sequences of the smaller dataset (17653). [file Image_1.TIF]

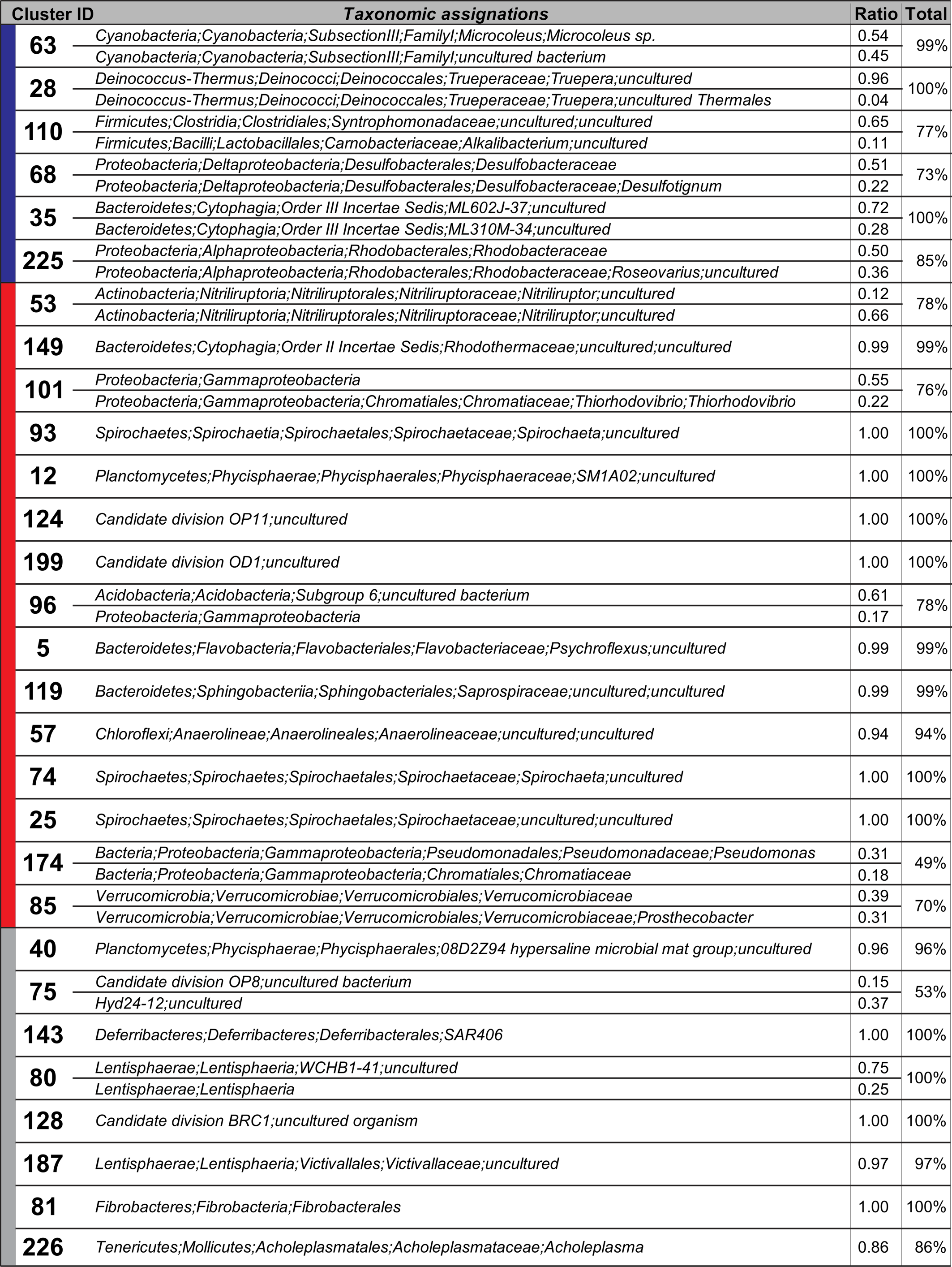

Supplement: FIGURE S2 — Taxonomic assignation of the clusters ID in the heatmap. Clusters ID numbers along with the major taxonomic assignations (see Materials and Methods), the contribution rate for a given assignation in the cluster, and the total percentage of sequences in the cluster represented with those taxonomic assignations. [file Image_2.TIF]
